# Pulmonary function of children with acute leukemia in maintenance phase of chemotherapy☆

**DOI:** 10.1016/j.rpped.2014.06.005

**Published:** 2014-12

**Authors:** Thalita Medeiros Fernandes de Macêdo, Tania Fernandes Campos, Raquel Emanuele de França Mendes, Danielle Corrêa França, Gabriela Suéllen da Silva Chaves, Karla Morganna Pereira Pinto de Mendonça

**Affiliations:** a Universidade Federeal do Rio Grande do Norte (UFRN), Natal, RN, Brazil; b Universidade Federal de Minas Gerais (UFMG), Belo Horizonte, MG, Brazil

**Keywords:** Child, Leukemia, Respiratory system, Respiratory muscles, Spirometry

## Abstract

**OBJECTIVE:**

The aim of this study was to assess the pulmonary function of children with acute leukemia.

**METHODS:**

Cross-sectional observational analytical study that enrolled 34 children divided into groups A (17 with acute leukemia in the maintenance phase of chemotherapy) and B (17 healthy children). The groups were matched for sex, age and height. Spirometry was measured using a spirometer Microloop Viasys^(r)^ in accordance with American Thoracic Society and European Respiratory Society guidelines. Maximal respiratory pressures were measured with an MVD300 digital manometer (Globalmed^(r))^. Maximal inspiratory pressures and maximal expiratory pressures were measured from residual volume and total lung capacity, respectively.

**RESULTS:**

Group A showed a significant decrease in maximal inspiratory pressures when compared to group B. No significant difference was found between the spirometric values of the two groups, nor was there any difference between maximal inspiratory pressure and maximal expiratory pressure values in group A compared to the lower limit values proposed as reference.

**CONCLUSION:**

Children with acute leukemia, myeloid or lymphoid, during the maintenance phase of chemotherapy exhibited unchanged spirometric variables and maximal expiratory pressure; However, there was a decrease in inspiratory muscle strength.

## Introduction

Leukemia, a malignant disease most frequently found in patients aged from 0 to 18 years old, represents 25-35% of all tumors in this population.^1^ Leukemia almost always presents in its acute form in children. Leukemia is classified according to cytology, immunohistochemistry and cytogenetics as acute lymphoblastic leukemia (ALL) and acute myeloid leukemia (AML).^2^ ALL represents 70-80% of the cases and AML represents approximately 15% of the cases.^3^

The chosen treatment for this neoplasia is chemotherapy, which can be used in conjunction with other therapies. The chemotherapy protocols last more than a year and a half.^4^ The treatment is divided into phases with the maintenance phase being the lengthiest period of the treatment. It is also the stage in which children already have greater clinical stability and have passed through the other stages.^4^

In the last four decades, continuous improvements in treatment results have been observed in children with this neoplasia. Due to enhanced prognosis, there is a need to consider the morbidity induced by treatment protocols. Studies report the following complications: secondary leukemia and complications in musculoskeletal, pulmonary, urinary, gastrointestinal, cardiac and nervous systems.^5^

Children treated for cancer face the risk of complications including pulmonary dysfunction.^6^ Pulmonary toxicity due to the chemotherapy or associated with radiotherapy may also result in interstitial lung injury during an initial stage until several months after the treatment and, in a late stage, the most common problem is lung fibrosis.^7^

It has been demonstrated that the use of high doses of cyclophosphamide, arabinosil cytosine, anthracyclines, dexamethasone and 6- thioguanine, medications used in the treatment of leukemia, as well as their combination, may lead to pulmonary toxicity and predispose to infections.^8^^,^ 9 High-doses of anthracycline may affect lung function causing congestive heart failure.^8^ Use of higher doses of arabinosil cytosine, anthracyclines, and cyclophosphamide intravenously have been associated with reduced lung volumes and capacities.^8^ Chemotherapy-induced lung fibrosis in children can remain asymptomatic for many years and may become symptomatic at any time.^9^

Studies report that the pulmonary function of individuals with leukemia may be altered^8^^,^ 10; however, it is not known whether these alterations are already present during chemotherapy or only at long-term. The role of physiotherapy in these patients is necessary to minimize the adverse effects of treatments. Accordingly, this study aimed at assessing the pulmonary function of children with acute leukemia during the maintenance phase of chemotherapy treatment and, thus, to identify whether the pulmonary function is already altered during the acute phase of the treatment. 

## Method

This cross-sectional observational analytical study was approved by the Research Ethics Committee of the Federal University of Rio Grande do Norte (no. 273/2008) and the LIGA Norte Riograndense Contra o Câncer (no. 185/185/2010 and 086/086/2011). Research was in accordance with the Declaration of Helsinki criteria.

The sample was composed of children aged between 5 and 12 years, diagnosed with acute leukemia and in the maintenance phase of chemotherapy treatment at 3 centers for childhood cancer in Rio Grande do Norte state, Northeast Brazil, (group A), as well as healthy school children, matched with group A for sex, age and height (group B). They were considered healthy when they did not have history of leukemia and/or acute or chronic diseases of the respiratory system. The criteria for assessment and classification in groups A and B are described below. 

To be included in the study, children could not display any of the following: diagnosis of cardiovascular or neuromuscular disease; diagnosis of chronic pulmonary disease on the standardized American Thoracic Society (ATS) and Division of Lung Diseases questionnaires ATS-DLD-78-C;^11^ respiratory infection in the previous two weeks,^12^ nausea or vomiting; thoracic deformity^13^ or recent thoracic or abdominal surgery^13^; hemoptysis, pneumothorax, cardiocirculatory instability^13^; pulmonary thromboembolism, cerebral, thoracic or abdominal aneurisms;^13^ recent upper airway, thoracic or abdominal trauma;^13^ acute middle ear problems;^13^ abdominal hernia;^13^ glaucoma, retinal detachment^13^ or recent eye surgery;^13^ neurological impairment,^12^^,^ 13 use of medication such as bronchodilators, anticholinergics, antihistamines and antileukotrienes.^12^

Participants who were unable to perform or understand any of the procedures were excluded, as well as those that: abandoned the study; had an acute respiratory tract disease during data collection; had been hospitalized for treatment; or missed classes or appointments at the outpatient facility during the assessment period.

Twenty-five children were treated at the above mentioned hospitals between January and September, 2011. Parents or legal guardians were informed about the study and gave their written consent. The parents of children of group B received an envelope containing the documentation required to take part in the study. In addition to parental consent, children participated only if they agreed to do so. A booklet containing appropriate language for the children's age range was used to inform them about the experiment.

All patients underwent initial assessment, which involved collecting personal, spirometric and maximal respiratory pressures data. Peripheral oxygen saturation, blood pressure and heart rate were monitored during evaluations. 

Spirometry was conducted using an MK8 Microloop Viasys portable digital spirometer (Cardinal Health U.K. 232 LTD). The device follows ATS and European Respiratory Society guidelines.^14^ The equipment was manually calibrated on a daily basis using a 3-liter syringe to ensure accuracy. A disposable mouthpiece and bactericidal filter were coupled to the spirometer. Spirometric measurements of the children were conducted according to ATS and European Respiratory Society norms for preschool children,^15^ for children 5 and 6 years old, and for those between the ages of 7 and 12.^14^ During the test all participants remained seated, using the nasal clip and with their heads in the neutral position. The children were instructed to breathe in as deeply as possible, pause for 1 to 2 seconds and then breathe out with maximum effort, continuing to exhale until the end of the test.^14^ Furthermore, maneuvers had to be free of coughing, air leaks, mouthpiece obstruction, valsalva maneuver, glottal closing, hesitation or new inspiration.

Children between the ages of 7 and 12 years had to exhibit a volume-time curve that showed no change in volume greater than or equal to 0.025l during the last second (plateau); satisfactory test time (in general 3 seconds in children up to 10 years old and 6 seconds in children older than 10). To ensure that forced expiratory volume in the first second (FEV_1_) was performed on a maximum effort curve, retro-extrapolated volume had to be 5% of forced vital capacity (FVC) or 0.150l, whichever was higher. At least 3 and at most 8 maneuvers were performed to obtain 3 acceptable ones (using the aforementioned criteria), with maximum difference of 0.150L (for FVC values above 1 liter) or 0.1L(for FVC values below 1 liter) between the two highest. The largest measures from the two tests were selected.^14^

The 5 and 6 years old were required to perform the following: flow-volume curves that showed a rapid increase up to peak flow; retro-extrapolated volume less than or equal to 80ml or less than 12.5% of FVC; at least 3 maneuvers, but with no maximum number; at least 2 acceptable maneuvers, in which the two highest FEV_1_ and FVC could not differ by more than 0.1L or 10%.^15^

A 1-minute rest period was given between each maneuver and subjects were provided with visual and verbal encouragement during assessment. Flow-volume and volume-time curves, performed at maximum effort, were analyzed after each maneuver. 

FEV_1_, FVC and peak expiratory flow values that could be extracted from different curves were selected from acceptable and reproducible curves, and the forced expiratory flow value between 25% and 75% of FVC (FEF_25-75%_) was selected from the curve with the highest sum of FVC and FEV_1._ 14

Respiratory muscle strength was performed 10 minutes after spirometry. Maximal inspiratory pressure (MIP) and maximal expiratory pressure (MEP) were measured according to the method proposed by Souza,^13^ using an MVD300 digital manometer (Globalmed(r), Porto Alegre - RS, Brazil), calibrated between -300 and +300 cmH2O, sensitive to each one-centimeter variation in water. The device was connected to a disposable biological filter, which was coupled to a flat rigid mouthpiece. The manometer was connected to a laptop that provided visual feedback. Participants also received verbal feedback during maneuvers. 

To measure MIP, subjects were instructed to breathe at tidal volume during three consecutive respiratory cycles and after the examiner`s command performed maximum expiration (approximately up to residual volume). They were then asked to execute maximum inspiration approximately up to total lung capacity.

Similar instructions were given to evaluate MEP, differing in that individuals first performed maximum inspiration, followed by maximum expiration. During this measurement, the examiner supported the participants` cheeks to ensure minimum loss of respiratory pressure due to complacency of the oral cavity.^16^ At most, 9 maneuvers were carried out for each maximal respiratory pressure,^17^ where at least three were acceptable (without air leaks and lasting at least 2 seconds) and 2 reproducible ones were performed (with values that did not differ by more than 10% of the highest value), the highest of which was used. Since the last measure could not be the highest, another one was taken if this occurred. 

A 1-minute rest period was given between each maneuver and 5 minutes between measurements of MIP and MEP. Children remained seated and wore a nasal clip during the entire test.

Since the manometer used produces a direct measure of peak pressure, sustained pressure was determined by analyzing the pressure versus time curve provided by the manometer software. Values were exported to the Microsoft Office Excel program and analyzed according to the protocol proposed by Borja.^18^

Statistical analysis was conducted with Statistical Package for the Social Science (SPSS) 17.0 software at a 5% significance level. The Shapiro-Wilk test was applied to verify data normality. Descriptive analysis was performed using means and standard deviations. 

The non-paired student`s t-test was used to compare variables between groups A and B. Considering that study power is defined as the capacity to demonstrate a statistically significant difference (or "effect"), the effect size was determined from Cohen's d calculation^19^ for comparisons between group A and B children.

## Results

Among the 25 children on the maintenance phase of chemotherapy, 6 were ineligible to take part in the study (3 had Down syndrome, 1 showed an enlarged heart (cardiomegaly) and 2 did not have parental consent). The 19 remaining children were assigned to group A. Two of them did not understand the examiner`s command. Concerning the leukemia, 88.2% of participants showed ALL and 11.8% AML. Group B was composed of 17 healthy school children matched with group A patients. Therefore, the final study sample consisted of 34 children, 24 boys and 10 girls (mean age of 6.83 ± 1.4 years and 6.2 ± 1.0 years, respectively).

The analysis of the anthropometric variables of group A and B subjects did not show significant difference between groups concerning the weight (*p=*0.60), height (*p=*0.88) and body mass index (*p=*0.44). Table 1 shows a comparative analysis of measures obtained by spirometry and manometry.

**Table 1:**
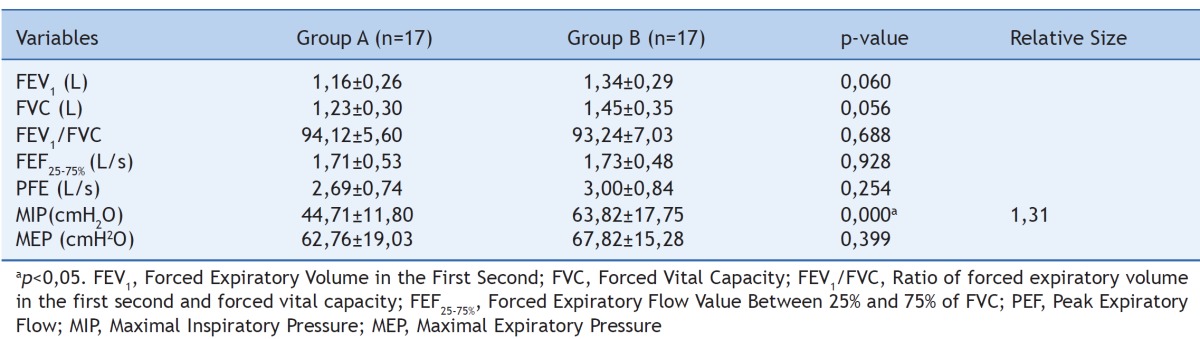
Spirometric variables and maximal respiratory pressures obtained in groups A and B; values of the mean, standard deviation, effect size and significance level.

The values for maximal respiratory pressures obtained among 7 years old and older children from group A were compared with the normal lower limits proposed by Borja.18 No significant difference was recorded on MIP and MEP values between group A and those proposed by Borja^18^ (*p=*0.96 and *p=*0.58, respectively).

Table 2 classifies the relative size of the statistical power of the study using Cohen's d calculation.^19^

**Table 2:**
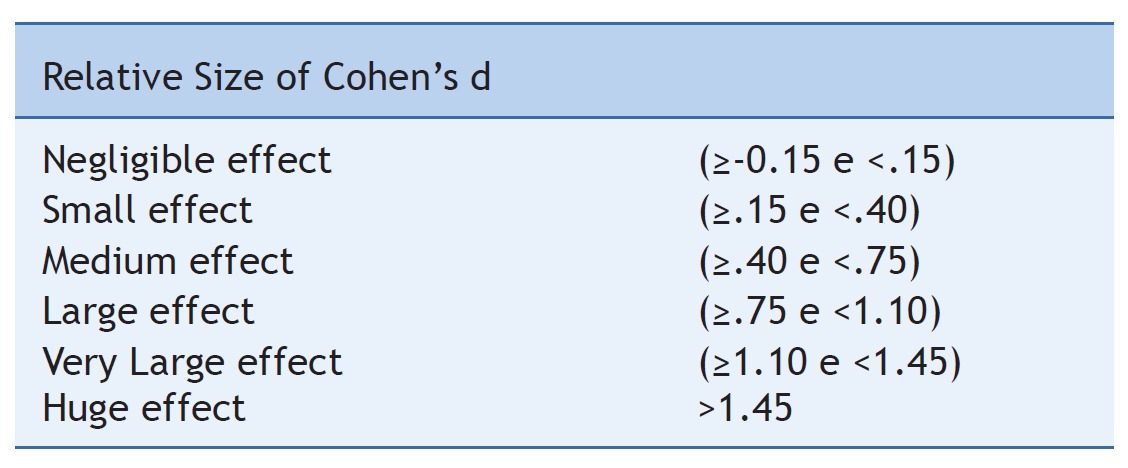
Classification of the relative size of the statistical power of the study using Cohen's d calculation19.

## Discussion

The assessment of pulmonary function in children with acute leukemia demonstrates that spirometric and MEP variables of the study sample are within the expected for healthy controls. By contrast, the MIP is decreased. We found no studies considering the immediate effects of chemotherapy on the pulmonary function of children with leukemia. Most studies reached a consensus regarding the delayed effects of chemotherapy on the spirometry of leukemia survivors.^6^^,^ 8 ^,^ 10

A group of authors^10^ has recently performed spirometric tests in 42 children with hematological oncology diseases, comparing data with healthy children. The authors reported that 19% of the children with cancer revealed severely limited air flow before treatment. They also observed that three years after the clinical treatment, half of these children recovered normal pulmonary function while the remainder exhibited a worsened pattern. Finally, of the 42 children assessed, 38 showeda lightly impaired air flow in the long term.

Other studies also showed consistent results regarding delayed impairment in spirometric variables after diagnosis of leukemia in childhood.^8^^,^ 20 In the first study,^8^ the authors performed spirometry in patients with acute lymphoid leukemia in childhood which were treated with different chemotherapy and radiotherapy protocols. After eight years, 61% of the sample had normal pulmonary function. Reduced pulmonary function was related to the young age and the use of more intensive protocols. In the second study,20 the researchers made a comparative analysis of three patient groups (chemotherapy, chemotherapy and radiotherapy and chemotherapy, radiotherapy and bone marrow transplant groups) 10 years after their acute myeloid leukemia treatment and observed that 20% of the patients treated with chemotherapy, radiotherapy and bone marrow transplant presented mild restrictive lung disorder.

Researchers from Egypt^6^ performed pulmonary tests in children who survived leukemia and lymphoma, observing that 25% of those treated with chemotherapy alone had pulmonary dysfunction. This percentage was higher than 70% among those who also required radiotherapy.

These studies show that deteriorated pulmonary function in patients with hematological cancer seems to be strongly related to more aggressive chemotherapy protocols and the addition of radiation and/or bone marrow transplant. From the present study, Group A, composed of children with acute leukemia on the maintenance phase of chemotherapy, did not undergo radiotherapy or bone marrow transplant, in addition to chemotherapy.This aspect, supported by the lack of scientific evidence of immediate spirometric alterations following chemotherapy alone, seems to justify the our findings. Therefore, during the maintenance phase of chemotherapy, children do not seem to present significant decrease in lung volumes and capacities when compared to healthy controls.

Loss of respiratory muscle strength can occur before reduced pulmonary function is detected, as seen in neuromuscular disorders.^21^ Macedo *et al* 22 assessed the maximal respiratory pressures of 14 children with acute leukemia and observed a decrease in MIP and in MEP in most children, according to normal values proposed by Wilson *et al* 23. Oliveira *et al* 24 assessed the MIP and MEP of children with acute leukemia, comparing them with measures obtained in healthy controls and found a significant reduction in MIP in children with acute leukemia, as shown in the present study. The lower limit of normal for maximal respiratory pressures has been used to confirm if a patient is suffering from respiratory muscle weakness.^25^ If the value obtained for maximal respiratory pressures is less than the lower limit of normal proposed, the chance of respiratory muscle weakness is 95%.^25^ Despite the significant reduction in MIP in group A children from the present study, this variable was higher than the lower limit of normal recently proposed for the studied age range.^18^ These findings suggest that the chance of these children being definitively diagnosed with inspiratory muscle weakness is at most 5%.

The study has its limitations. The wide divergence concerning treatment protocols used in earlier studies and the difficulty on finding pulmonary function data during the acute phase of chemotherapy treatment limited the discussion of our findings. A further limitation is the absence of more accurate information on the physical activity levels of the participating children. Moreover, the impossibility to analyze the pulmonary function of children with the two studied types of leukemia sepatately, as well as non-precise description of the medication and its dosage used in the leukemia treatment impair a more detailed discussion of the findings. 

Currently, the evaluation of lung function is not yet part of the routine monitoring of outpatients with blood cancers. Although the literature indicates an improvement in survival of children with leukemia subjected to more advanced treatment protocols, there is still little information about respiratory evaluation of these patients.^5^ Thus, this pioneer study may add information about the lung function effects of these treatments in children with acute leukemia. The identification of these effects may guide a best physical therapy care to these children.

Finally, the spirometric variables of children with acute leukemia did not change during the chemotherapy maintenance phase. Although MEP of children with leukemia did not differ significantly from that of the healthy controls, MIP declined in this population. As a result, physiotherapy could be performed in an attempt to preserve muscle strength, minimizing consequent effects of respiratory muscle weakness which could have an effect on quality of life in children with cancer.
