# Supplementary material for: Pulmonary function of children with acute leukemia in maintenance phase of chemotherapy
Source: Rev Paul Pediatr. 2014 Dec;32(4):320–5. doi: 10.1016/j.rpped.2014.06.005 (PMC4311785; doi:10.1016/j.rpped.2014.06.005)
Supplement: Translation

# Translation: Função pulmonar de crianças com leucemia aguda na fase de manutenção da quimioterapia☆

**Authors:** Thalita Medeiros Fernandes de Macêdo, Tania Fernandes Campos, Raquel Emanuele de França Mendes, Danielle Corrêa França, Gabriela Suéllen da Silva Chaves, Karla Morganna Pereira Pinto de Mendonça

**Affiliations:** a Universidade Federal do Rio Grande do Norte (UFRN), Natal, RN, Brasil; b Universidade Federal de Minas Gerais (UFMG), Belo Horizonte, MG, Brasil

**Keywords:** Criança, Leucemia, Sistema respiratório, Músculos respiratórios, Espirometria

## Abstract

**OBJETIVO:**

O objetivo desse estudo foi avaliar a função pulmonar de crianças com leucemia aguda.

**MÉTODOS:**

Trata-se de um estudo observacional do tipo analítico transversal com 34 crianças, divididas nos grupos A (17 crianças com leucemia aguda na fase de manutenção do tratamento quimioterápico) e B (17 crianças saudáveis). Os grupos foram pareados em relação ao sexo, idade e altura. A espirometria foi mensurada utilizando um espirômetro Microloop Viasys^(r)^, de acordo com as recomendações da *American Thoracic Society* e *European Respiratory Society*. As pressões respiratórias máximas foram mensuradas utilizando um manovacuômetro digital MVD300 (Globalmed(r)). As pressões inspiratória máxima e expiratória máxima foram mensuradas a partir do volume residual e da capacidade pulmonar total, respectivamente.

**RESULTADOS:**

O grupo A apresentou diminuição significativa da pressão inspiratória máxima quando comparado ao grupo B. Não foram observadas diferenças entre os dados espirométricos dos dois grupos avaliados, bem como entre os valores de pressão inspiratória máxima e pressão expiratória máxima do grupo A com os limites inferiores propostos como referência.

**CONCLUSÃO:**

As crianças com leucemia aguda, linfoide ou mieloide não apresentam mudança das variáveis espirométricas e da pressão expiratória máxima durante o período de manutenção do tratamento quimioterápico; no entanto, há uma diminuição da pressão inspiratória máxima.

## Introdução

A leucemia, uma doença maligna mais frequentemente encontrada em pacientes com idade de 0 a 18 anos de idade, representa 25-35% de todos os tumores nessa população.^1^ A leucemia aparece quase sempre na sua forma aguda em crianças. A leucemia é classificada de acordo com a citologia, imuno-histoquímica e citogenética como leucemia linfocítica aguda (LLA), e leucemia mieloide aguda (LMA).^2^ A LLA representa 70-80% dos casos, e a LMA, cerca de 15% dos casos.^3^

O tratamento de escolha para essa neoplasia é a quimioterapia, que pode ser utilizada em conjunto com outras terapias. Os protocolos de quimioterapia duram mais que um ano e meio.^4^ O tratamento é dividido em fases, com a fase de manutenção sendo o período mais extenso do tratamento. É também a fase em que as crianças já têm uma maior estabilidade clínica e passaram por outras etapas.^4^

Nas últimas quatro décadas, melhorias contínuas nos resultados do tratamento foram observadas em crianças com essa neoplasia. Por conta de um melhor prognóstico, há a necessidade de considerar a morbidade induzida por protocolos de tratamento. Estudos relatam as seguintes complicações: leucemia secundária e complicações nos sistemas: musculoesquelético, pulmonar, urinário, gastrointestinal, cardíaco e nervoso.^5^

As crianças recebendo tratamento para câncer enfrentam o risco de complicações, incluindo disfunção pulmonar.^6^ A toxicidade pulmonar em consequência da quimioterapia ou associada à radioterapia pode também resultar em lesão pulmonar intersticial durante uma fase inicial até vários meses após o tratamento e, em uma fase tardia, o problema mais comum é a fibrose pulmonar.^7^

Tem sido demonstrado que o uso de doses elevadas de ciclofosfamida, arabinosil citosina, antraciclinas, dexametasona e 6-tioguanina, os medicamentos utilizados no tratamento da leucemia, bem como a sua combinação, podem levar à toxicidade pulmonar e predispor a infecções.^8^^,^ 9 Altas doses de antraciclina podem afetar a função pulmonar, causando insuficiência cardíaca congestiva.^8^ A utilização de doses mais elevadas de citosina arabinosil, antraciclinas, e ciclofosfamida por via intravenosa tem sido associada a volume e capacidade pulmonares reduzidos.^8^ A fibrose pulmonar induzida por quimioterapia em crianças pode permanecer assintomática por muitos anos e pode tornar-se sintomática a qualquer momento.^9^

Estudos relatam que a função pulmonar de indivíduos com leucemia pode estar alterada;^8^^,^ 10 no entanto, não se sabe se essas alterações já estão presentes durante a quimioterapia ou apenas em longo prazo. O papel da fisioterapia nesses pacientes é necessário para minimizar os efeitos adversos dos tratamentos. Assim, esse estudo teve como objetivo avaliar a função pulmonar de crianças com leucemia aguda durante a fase de manutenção do tratamento quimioterápico e, portanto, identificar se a função pulmonar já está alterada durante a fase aguda do tratamento. 

## Método

Esse estudo observacional do tipo analítico transversal foi aprovado pelo Comitê de Ética em Pesquisa da Universidade Federal do Rio Grande do Norte (n º 273/2008) e pela Liga Norte Riograndense Contra o Câncer (n º 185/185/2010 e 086/086/2011). A pesquisa se ​​deu em conformidade com os critérios da Declaração de Helsinque. 

A amostra foi composta por crianças com idades entre 5 e 12 anos, com diagnóstico de leucemia aguda e na fase de manutenção do tratamento quimioterápico em três centros de câncer infantil no Rio Grande do Norte, Nordeste do Brasil, (grupo A), bem como por crianças saudáveis em idade escolar, pareadas com o grupo A para sexo, idade e altura (grupo B). Elas foram consideradas saudáveis ​​quando não apresentavam histórico de leucemia e/ou doenças agudas ou crônicas do sistema respiratório. Os critérios para a avaliação e classificação dos grupos A e B encontram-se descritos abaixo. 

Para serem incluídas no estudo, as crianças não poderiam apresentar o seguinte: diagnóstico de doença cardiovascular ou neuromuscular; diagnóstico de doença pulmonar crônica de acordo com os questionários-padrão da American Thoracic Society (ATS) e da Divisão de Doenças de Pulmão ATS-DLD-78-C;^11^ infecção respiratória nas duas semanas anteriores,^12^ náusea ou vômito; deformidade torácica^13^ ou cirurgia torácica/abdominal recente;^13^ hemoptise, pneumotórax, instabilidade cardiocirculatória;^13^ tromboembolismo pulmonar, aneurismas cerebral, torácico ou abdominal;^13^ trauma recente torácico, abdominal ou das vias aéreas superiores;^13^ problemas agudos do ouvido médio;^13^ hérnia abdominal;^13^ glaucoma, descolamento de retina^13^ ou cirurgia ocular recente;^13^ comprometimento neurológico,^12^^,^ 13 uso de medicamentos como broncodilatadores, anticolinérgicos, anti-histamínicos e antileucotrienos.^12^

Os participantes que não puderam realizar ou compreender qualquer um dos procedimentos foram excluídos, bem como aqueles que: abandonaram o estudo; apresentaram uma doença aguda do trato respiratório durante a coleta de dados; haviam sido hospitalizados para tratamento; ou perderam aulas ou consultas no ambulatório durante o período de avaliação. 

Vinte e cinco crianças foram tratadas nos hospitais mencionados acima, entre janeiro e setembro de 2011. Os pais ou responsáveis legais ​​foram informados sobre o estudo e deram seu consentimento por escrito. Os pais das crianças do grupo B receberam um envelope contendo a documentação necessária para participar no estudo. Além do consentimento dos pais, as crianças participaram somente com a sua própria concordância. Um livreto contendo linguagem apropriada para a faixa etária das crianças foi utilizado para informá-las sobre o experimento. 

Todos os pacientes foram submetidos à avaliação inicial, que envolveu a coleta de dados pessoais, pressões respiratórias máximas e dados de espirometria. A taxa de saturação periférica de oxigênio, pressão arterial e frequência cardíaca foram monitoradas durante as avaliações. 

A espirometria foi realizada utilizando um espirômetro digital portátil MK8 Microloop Viasys (Cardinal Health, Londres). O dispositivo segue as diretrizes da ATS e da Sociedade Respiratória Europeia.^14^ O equipamento foi calibrado manualmente, diariamente, utilizando uma seringa de 3 litros para garantir a precisão. Um bocal descartável e filtro bactericida foram acoplados ao espirômetro. As medições espirométricas das crianças foram conduzidas de acordo com as normas da ATS e da Sociedade Respiratória Europeia para crianças pré-escolares,^15^ para crianças de 5 e 6 anos de idade, e para aquelas com idades entre 7 e 12 anos.^14^ Durante o teste, todos os participantes permaneceram sentados, usando o clipe nasal e com as suas cabeças em posição neutra. As crianças foram instruídas a respirar o mais profundamente possível, pausando por 1 a 2 segundos e depois expirando com o máximo esforço, continuando a exalar até o final do teste.^14^ Além disso, as manobras tinham que estar livres de tosse, vazamentos de ar, obstrução do bocal, manobra de Valsalva, fechamento da glote, hesitação ou uma nova inspiração. 

As crianças com idades entre 7 e 12 anos tiveram que apresentar uma curva de volume-tempo que não mostrasse nenhuma mudança no volume maior ou igual a 0,025l durante o último segundo (platô); tempo de teste satisfatório (em geral 3 segundos em crianças até aos 10 anos de idade e 6 segundos em crianças com mais de 10). Para garantir que o volume expiratório forçado no primeiro segundo (VEF_1_) tivesse sido realizado em uma curva de esforço máximo, o volume retro-extrapolado tinha que ser 5% da capacidade vital forçada (CVF) ou 0,150l, se esse fosse superior. Pelo menos três e no máximo oito manobras foram realizadas para obter três aceitáveis ​​(usando os critérios acima mencionados), com a diferença máxima de 0,150L (para CVF acima de 1 litro) ou 0,1L (para CVF abaixo de 1 litro) entre os dois maiores valores. Foram selecionadas as maiores medidas dos dois testes.^14^

Para as crianças de 5 e 6 anos de idade, foi solicitado que executassem o seguinte: curvas de fluxo-volume que mostrassem um aumento rápido até o pico de fluxo; volume retro-extrapolado inferior ou igual a 80mL, ou menos de 12,5% de CVF; pelo menos três manobras, mas sem um número máximo; pelo menos duas manobras aceitáveis​​, em que os dois maiores VEF_1_ e CVF não diferissem em mais do que 0,1L ou 10%.^15^

Foi dado um período de descanso de 1 minuto entre cada manobra e foram dados incentivos visuais e verbais aos indivíduos durante a avaliação. As curvas fluxo-volume e volume-tempo, realizadas em esforço máximo, foram analisadas após cada manobra. 

Os valores VEF_1_, CVF e de pico de fluxo expiratório que puderam ser extraídos a partir de diferentes curvas foram selecionados a partir de curvas aceitáveis ​​e reprodutíveis, e o valor do fluxo expiratório forçado entre 25% e 75% da CVF (FEF_25-75%_) foi selecionado a partir da curva com a maior soma de CVF e VEF_1_.^14^

A força muscular respiratória foi executada 10 minutos após a espirometria. A pressão inspiratória máxima (PImáx) e pressão expiratória máxima (PEmáx) foram medidas de acordo com o método proposto por Souza,^13^ utilizando um manômetro digital MVD300 (Globalmed(r), Rio Grande do Sul, Brasil), calibrado entre -300 e +300 cmH_2_O, sensível a cada variação de um centímetro de água. O dispositivo foi ligado a um filtro biológico descartável que foi acoplado a um bocal plano e rígido. O manômetro foi conectado a um *laptop* que fornecia *feedback* visual. Os participantes também receberam *feedback* verbal durante as manobras. 

Para medir a PImáx, os indivíduos foram orientados a respirar no volume tidal durante três ciclos respiratórios consecutivos e, após o comando do examinador, realizar expiração máxima (aproximadamente até o volume residual). Eles foram, então, solicitados a executar a inspiração máxima até aproximadamente a capacidade pulmonar total. 

Instruções semelhantes foram dadas para avaliar PEmáx, com a diferença que os indivíduos primeiro realizaram uma inspiração máxima, seguida de expiração máxima. Durante essa medição, o examinador apoiou as bochechas dos participantes para garantir a perda mínima de pressão respiratória em consequência da complacência da cavidade oral.^16^ Um máximo de nove manobras foram realizadas para cada pressão respiratória máxima,^17^ onde pelo menos três eram aceitáveis ​​(sem vazamentos e durando pelo menos 2 segundos) e duas reprodutíveis foram realizadas (com valores não diferindo em mais de 10% do valor mais alto), sendo que o maior valor foi usado. Como a última medida não podia ser a mais alta, outra medida foi obtida quando isso ocorria. 

Foram dados um período de descanso de 1 minuto entre cada manobra e um período de 5 minutos entre as medidas de PImáx e PEmáx. As crianças permaneceram sentadas e usaram um clipe nasal durante todo o teste. 

Como o manômetro utilizado produz uma medida direta da pressão de pico, a pressão contínua foi determinada através da análise da pressão *versus* a curva de tempo fornecida pelo software do manômetro. Os valores foram exportados para o programa Microsoft Office Excel e analisados ​​de acordo com o protocolo proposto por Borja.^18^

A análise estatística foi realizada com o software *Statistical Package for Social Science*s (SPSS) v. 17.0, com um nível de significância de 5%. O teste de Shapiro-Wilk foi aplicado para verificar a normalidade dos dados. A análise descritiva foi realizada por meio de médias e desvios-padrão. 

O teste *t* de Student não pareado foi utilizado para comparar as variáveis ​​entre os grupos A e B. Considerando que o poder do estudo é definido como a capacidade de demonstrar uma diferença estatisticamente significativa (ou "efeito"), o tamanho do efeito foi determinado a partir do cálculo d de Cohen^19^ para comparações entre os grupos de crianças A e B. 

## Resultados

Entre as 25 crianças na fase de manutenção da quimioterapia, seis eram inelegíveis para participar no estudo (três tinham síndrome de Down, uma mostrou aumento do coração (cardiomegalia) e duas não tiveram o consentimento dos pais). As 19 crianças restantes foram alocadas ao grupo A. Duas delas não entenderam o comando do examinador. Em relação à leucemia, 88,2% dos participantes tinham LLA e 11,8%, LMA. O grupo B foi composto por 17 escolares saudáveis ​​pareados com os pacientes do grupo A. Portanto, a amostra final do estudo consistiu de 34 crianças, 24 meninos e 10 meninas (idade média de 6,83 ± 1,4 anos e 6,2 ± 1,0 anos, respectivamente). 

A análise das variáveis ​​antropométricas dos indivíduos dos grupos A e B não apresentou diferença significativa entre os grupos em relação ao peso (*p=*0,60), altura (*p=*0,88) e índice de massa corporal (*p=*0,44). A tabela 1 mostra uma análise comparativa das medidas obtidas pela espirometria e manometria. 

**Tabela 1:** Variáveis espirométricas e pressões respiratórias máximas obtidas nos grupos A e B; valores médios, desvio-padrão, tamanho do efeito e nível de significância

Os valores das pressões respiratórias máximas obtidas entre crianças de 7 anos e crianças mais velhas do grupo A foram comparadas com os limites inferiores normais propostos por Borja.^18^ Nenhuma diferença significativa foi registrada entre os valores de PImáx e PEmáx do grupo A e os propostos por Borja^18^ (*p=*0,96 e *p=*0,58, respectivamente). 

A tabela 2 classifica o tamanho relativo do poder estatístico do estudo usando o cálculo d de Cohen.^19^

**Tabela 2:** Classificação do tamanho relativo do poder estatístico do estudo usando cálculo do d de Cohen19

## Discussão

A avaliação da função pulmonar em crianças com leucemia aguda demonstra que as variáveis ​​espirométricas e PEmáx da amostra do estudo estão dentro do esperado para os controles saudáveis. A PImáx, em contraste, está reduzida. Não encontramos estudos considerando os efeitos imediatos da quimioterapia sobre a função pulmonar de crianças com leucemia. A maioria dos estudos chegou a um consenso sobre os efeitos tardios da quimioterapia sobre a espirometria de sobreviventes da leucemia.^6^^,^ 8 ^,^ 10

Um grupo de autores^10^ realizou recentemente testes de espirometria em 42 crianças com doenças oncológicas hematológicas, comparando os dados com crianças saudáveis. Os autores relataram que 19% das crianças com câncer apresentaram fluxo de ar severamente limitado antes do tratamento. Eles também observaram que, três anos após o tratamento clínico, metade dessas crianças recuperou a função pulmonar normal, enquanto o restante exibiu um padrão piorado. Finalmente, das 42 crianças avaliadas, 38 mostraram fluxo de ar levemente prejudicado em longo prazo. 

Outros estudos também mostraram resultados consistentes com deficiências tardias nas variáveis ​​da espirometria após o diagnóstico de leucemia na infância.^8^^,^ 20 No primeiro estudo,^8^ os autores realizaram a espirometria em pacientes com leucemia linfoide aguda na infância, que foram tratados com diferentes protocolos de quimioterapia e radioterapia. Depois de oito anos, 61% da amostra apresentou função pulmonar normal. Função pulmonar reduzida foi relacionada com a pouca idade e a utilização de protocolos mais intensivos. No segundo estudo,^20^ os pesquisadores fizeram uma análise comparativa dos três grupos de pacientes (quimioterapia, quimioterapia e radioterapia, e quimioterapia, radioterapia e grupos de transplante de medula óssea) 10 anos depois do tratamento da leucemia mielóide aguda, e observaram que 20% dos pacientes tratados com quimioterapia, radioterapia e transplante de medula óssea apresentaram distúrbio ventilatório restritivo leve. 

Pesquisadores do Egito^6^ realizaram testes pulmonares em crianças que sobreviveram à leucemia e ao linfoma, observando que 25% dos pacientes tratados apenas com quimioterapia tinham disfunção pulmonar. Esse percentual foi superior a 70% entre aqueles que também exigiram radioterapia. 

Esses estudos mostram que a deterioração da função pulmonar em pacientes com câncer hematológico parece estar fortemente relacionada com os protocolos mais agressivos de quimioterapia e a adição de radiação e/ou transplante de medula óssea. No presente estudo, o Grupo A, composto por crianças com leucemia aguda na fase de manutenção da quimioterapia, não foi submetido a transplante de medula óssea ou radioterapia, em adição à quimioterapia. Esse aspecto, apoiado pela falta de evidência científica de alterações espirométricas imediatamente após a quimioterapia isolada, parece justificar os nossos achados. Portanto, durante a fase de manutenção quimioterápica, as crianças não parecem apresentar redução significativa nos volumes e capacidades pulmonares, quando comparados com os controles saudáveis​​. 

A perda de força muscular respiratória pode ocorrer antes que a função pulmonar reduzida seja detectada, conforme observado em doenças neuromusculares.^21^ Macedo *et al* 22 avaliaram as pressões respiratórias máximas de 14 crianças com leucemia aguda e notaram uma diminuição na PImáx e PEmáx na maioria das crianças, de acordo com os valores normais propostos por Wilson *et al*.^23^ Oliveira *et al* 24 avaliaram a PImáx e PEmáx de crianças com leucemia aguda, comparando-as com medidas obtidas em controles saudáveis, ​​e encontraram uma redução significativa na PImáx em crianças com leucemia aguda, como mostrado no presente estudo. O limite inferior do normal para as pressões respiratórias máximas tem sido utilizado para confirmar se um paciente está sofrendo de fraqueza muscular respiratória.^25^ Se o valor obtido para as pressões respiratórias máximas é menor que o limite inferior da normalidade proposta, a chance de fraqueza muscular respiratória é de 95%.^25^ Apesar da redução significativa da PImáx nas crianças do grupo A do presente estudo, esta variável foi maior do que o limite inferior da normalidade recentemente proposto para a faixa etária estudada.^18^ Esses resultados sugerem que a chance dessas crianças serem definitivamente diagnosticadas com fraqueza muscular inspiratória é no máximo de 5%. 

O estudo tem suas limitações. A grande divergência sobre os protocolos de tratamento utilizados em estudos anteriores e a dificuldade em encontrar dados de função pulmonar durante a fase aguda do tratamento quimioterápico limitaram a discussão de nossos resultados. Outra limitação é a ausência de informações mais precisas sobre os níveis de atividade física das crianças participantes. Além disso, a impossibilidade de analisar a função pulmonar de crianças com os dois tipos de leucemia estudados de forma separada, bem como a descrição não-precisa do medicamento e a sua dosagem utilizada no tratamento da leucemia prejudicam uma discussão mais pormenorizada dos resultados. 

Atualmente, a avaliação da função pulmonar ainda não faz parte do monitoramento de rotina de pacientes ambulatoriais com câncer hematológico. Embora a literatura indique uma melhora na sobrevida de crianças com leucemia submetidas a protocolos de tratamento mais avançados, ainda há pouca informação sobre a avaliação do sistema respiratório desses pacientes.^5^ Assim, esse estudo pioneiro pode adicionar informações sobre os efeitos desses tratamentos na função pulmonar em crianças com leucemia aguda. A identificação desses efeitos pode definir qual a melhor fisioterapia para essas crianças. 

Finalmente, as variáveis ​​espirométricas de crianças com leucemia aguda não se alteraram durante a fase de manutenção da quimioterapia. Embora a PEmáx de crianças com leucemia não tenha diferido significativamente em relação à dos controles saudáveis​​, a PImáx diminuiu nessa população. Como resultado, a fisioterapia pode ser realizada como uma tentativa de preservar a força muscular, minimizando os consequentes efeitos de fraqueza muscular respiratória, que podem ter impacto sobre a qualidade de vida de crianças com câncer.
